# Supratentorial vs infratentorial posterior calvarial distraction osteogenesis for the increase of ICV in children with syndromic or multi-suture craniosynostosis: a retrospective cohort study

**DOI:** 10.1007/s00381-021-05064-4

**Published:** 2021-02-05

**Authors:** Jack Sharman, Desiderio Rodrigues, Simon McGuirk, Mohini Panikkar, Hiroshi Nishikawa, Steve Dover, Martin Evans, Nicholas White

**Affiliations:** 1 The University of Birmingham Medical School, Birmingham, UK; 2 Department of Neurosurgery, Birmingham Children’s Hospital, Birmingham, UK; 3 Department of Interventional Radiology, Birmingham Children’s Hospital, Birmingham, UK; 4 The Royal Shrewsbury Hospital, Shrewsbury, UK; 5 Department of Craniofacial Surgery, Birmingham Children’s Hospital, Birmingham, UK

**Keywords:** Craniosynostosis, PCD, Supratentorial, Infratentorial

## Abstract

**Purpose:**

Craniosynostosis is the premature and pathological fusion of calvarial sutures. One modality of surgical treatment of syndromic craniosynostosis is posterior calvarial distraction (PCD). This can be either supratentorial or infratentorial. Currently, supratentorial PCD may be regarded as safer but produces a smaller increase in calvarial volume compared to infratentorial PCD. This study quantifies and compares the effectiveness of supratentorial and infratentorial PCD to help guide surgical decision-making.

**Methods:**

The CT and/or MRI scans of 47 cases of craniosynostosis who underwent PCD from the Birmingham Children’s Hospital (BCH) were converted to sagittal series multi-planar reformatted (MPR) scans for the manual calculation of ICV. The 47 cases were classified as having undergone either supratentorial or infratentorial PCD using lateral plain film radiographs, with 28 and 32 pairs of pre- and post-operative CT/MRI scans reviewed respectively.

**Results:**

A statistically significant difference between supratentorial and infratentorial PCD was observed for the increase in supratentorial volume (STV) (*P* = 0.0458) and total intracranial volume (TICV) (*P* = 0.0437), but not for the increase in infratentorial volume (ITV) (*P* = 0.0697). The relationship for each volume trended towards convergence but was not achieved before the physical limit of 30 mm distraction had been reached. Intraclass correlation coefficient values for agreement of MRI and CT scans for STV, ITV and total ICV were 0.852, 0.864 and 0.854 respectively.

**Conclusion:**

Our evidence suggests that supratentorial PCD is more effective for increasing ICV in a clinical setting. CT and MRI imaging modalities are acceptably clinically interchangeable for calculating ICV in craniosynostosis.

## Introduction

Craniosynostosis describes the premature fusion of one or more calvarial sutures [1]. This leads to craniocephalic disproportion resulting in functional and aesthetic sequelae [2, 3]. Functionally, raised intracranial pressure (ICP) manifests as developmental delay, seizures and hind-brain herniation, predominantly in syndromic or multi-suture craniosynostosis [4]. Uncertainty remains regarding how extensive craniosynostosis causes raised ICP. Evidence has shown that before 11 months of age those patients with craniosynostosis and raised ICP have greater ICVs than their age-matched peers [5]. The prevailing theory argues that craniocephalic disproportion causes aberrant venous drainage resulting in raised ICP [2]. Aesthetically, limited expansion at specific calvarial sutures results in asymmetrical skull growth and abnormal head shape [6]. Posterior calvarial distraction (PCD) may be used to normalize ICP and rectify head shape with controlled calvarial expansion [7]. This can be performed supratentorially or infratentorially, the distinction being the position of the inferior osteotomy relative to the torcular shelf and subsequently the tentorium cerebelli [8]. It has been regarded that infratentorial PCD confers greater outcomes, due to the greater surface area of mobile calvarium, thereby justifying the added risk [9]. Supratentorial PCD involves the inferior osteotomy crossing just the superior sagittal sinus at the confluence of sinuses, whilst infratentorial PCD risks rupturing the occipital venous sinus and both transverse dural venous sinuses whilst also posing greater risk to the medulla oblongata as the osteotomy approaches the foramen magnum; the prone and flexed nature of the patient’s neck intraoperatively widens the distance between the occipital bone and C1, thereby posing greater intraoperative risk [10]. Supratentorial PCD may be chosen over infratentorial PCD at the BCH due to patient-specific factors like anatomy and severity of craniosynostosis, and surgeon preference. The aim of this study is to elucidate which, if either, of supratentorial and infratentorial PCD is more effective at increasing ICV for each unit distance of distraction. It is intended that the results of this study help guide future surgical decision-making. An additional aim of this research is to evaluate the clinical interchangeability of CT and MRI imaging for calculating ICV in children with craniosynostosis. It is envisioned that this will further inform paediatric imaging decisions in cases of craniosynostosis to prevent, or justify, additional radiation exposure in young children.

## Methods

### Literature review

A search of MEDLINE (1946–2019), EMBASE (1947–2019), the Cochrane Library of Systematic Reviews (1996–2019), NICE Evidence Search and Google Scholar was performed using the following search terms: “Craniosynostosis”, “Posterior Distraction”, “Posterior Calvarial Distraction”, “Supratentorial”, “Infratentorial”, “ICV”, “Intracranial Volume” and “Torcula” with AND/OR Boolean operators. The initial search returned 214 papers, though NICE, Cochrane and EMBASE provided no search results. After the removal of 34 duplicates and review of all remaining paper titles for relevance (80 papers focused on torcular pathology, 54 papers were excluded for focusing on individual craniosynostotic syndromes and 44 papers were excluded for irrelevance to our clinical question), two papers remained. Neither publication focused on supratentorial or infratentorial PCD. Therefore, no papers specifically relevant to comparing supratentorial and infratentorial PCD were identified in our literature review.

### Patient selection

Seventy-two consecutive cases of craniosynostosis treated with PCD at the Birmingham Children’s Hospital (BCH) from 2006 to 2018 were identified. All cases were treated by the same craniofacial team. Of these 72 cases, 18 were excluded due to insufficient imaging (either scans had not been recorded or cases were too recent to have had follow-up CT/MRI scans). The remaining 54 cases were imported from IMPAX 6.5.2.2016 to OsiriX on Mac where a preliminary sagittal multi-planar reformat (MPR) conversion was conducted to gauge suitability for the study. Subsequently, six cases were excluded for having MPR imaging which fell below the threshold of diagnostic quality for this study. This was due to either poor image resolution (one case) or images not including the full bi-parietal distance (five cases). Due to structural asymmetry in the heads of craniosynostotic children, we could not extrapolate data from cases with an incomplete bi-parietal distance. From the remaining 48 cases, one was excluded for being the second episode of PCD for a patient; only the primary distraction was considered for this study. Forty-seven cases of craniosynostosis treated with PCD remained, encompassing 28 pairs of pre- and post-operative CT scans and 32 pairs of pre- and post-operative MRI scans with 13 cases having both paired CT and MRI scans. Seventeen and 21 cases of supratentorial and infratentorial PCD respectively displayed signs of raised ICP (any combination of ICP bolt measurements, developmental delays, headaches, papilloedema, Chiari malformation, image-based evidence or syrinx). The remaining cases, without indication of raised ICP, still showed multi-suture or pansynostosis, indicating PCD as an intervention.

### Determining PCD status

**Fig. 1:**
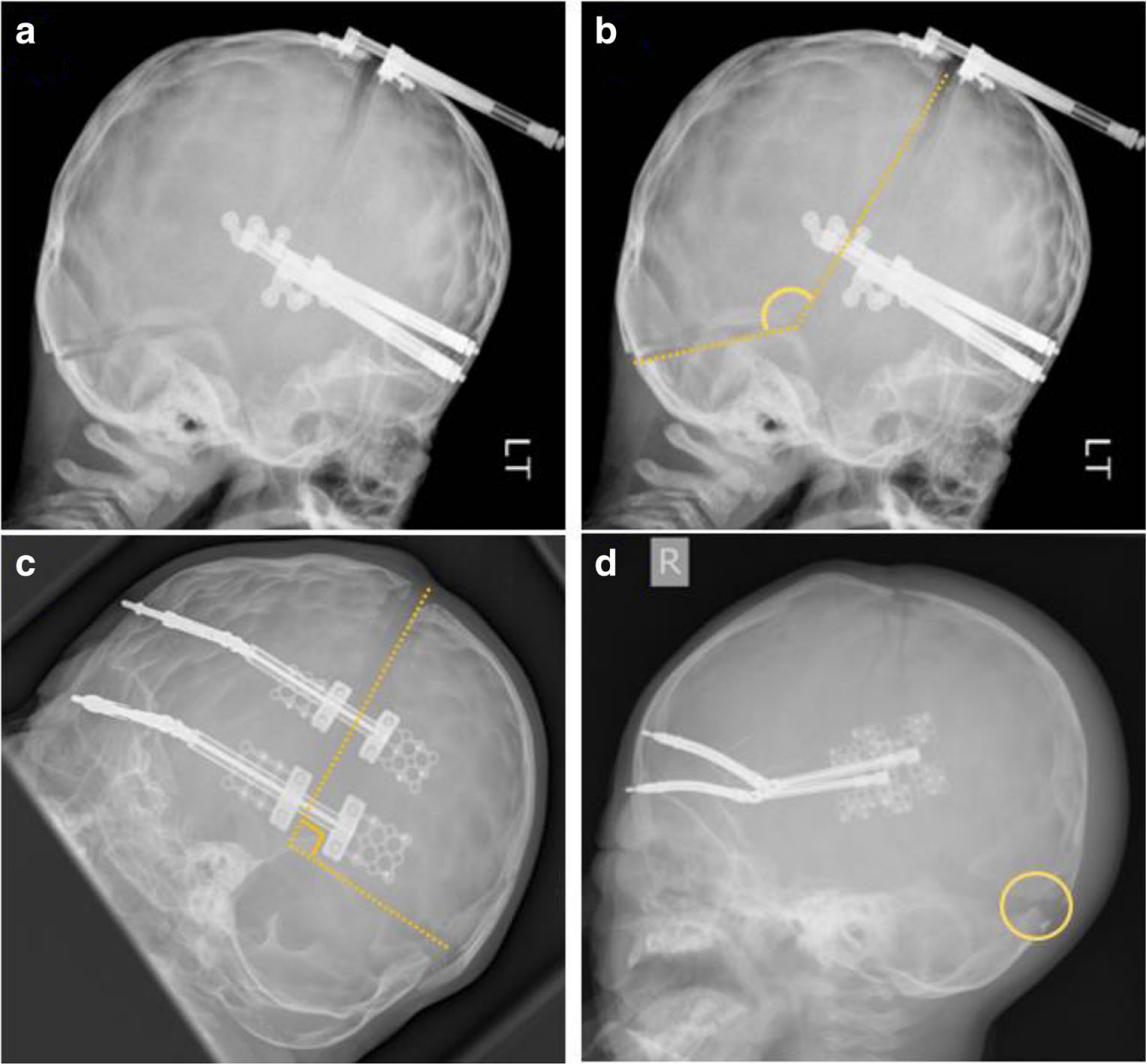
**a** A post-operative lateral plain film radiograph of a child, included in this study, having undergone infratentorial PCD. **b** A duplicate of panel **a** highlighting the osteotomies and the angle between them (yellow), confirming this child underwent infratentorial PCD. **c** A post-operative lateral plain film radiograph of a child, included in this study, having undergone supratentorial PCD—note the angle of the osteotomies (yellow) is approximately 90° and significantly different from that of panel **b**. **d** A post-operative lateral plain film radiograph of a child, included in this study, having undergone supratentorial PCD—note the torcular shelf viewable beneath the inferior osteotomy (yellow circle)

PCD status was determined from surgical notes specifying the variation of PCD, and immediately-post-operative lateral plain film radiographs. All 47 plain film radiographs were assessed by two independent reviewers, blinded to one another’s analysis. Criteria for determining PCD status from imaging were the angle of the osteotomies at the asterion and the position of the posterior-most aspect of the inferior osteotomy relative to the torcular shelf (where viewable) (Fig. 1). Discrepancies between the judgements of the two reviewers (2/47 cases) were adjudicated over by the consultant surgeon who performed the procedure and compared to patient notes. This data was kept separate from volume measurement data for the collection period to prevent unconscious bias.

### Volume measurement

The volume measurement dataset was randomised prior to analysis, so as to analyse the scans non-chronologically (in contrast to the chronological order, we analysed lateral films), to minimize the risk of information bias. The lateral boundaries of the sagittal MPR conversion were set at the outer table of the temporal bone and the slice thickness was set at 5 mm. This reduced the slice number from 40–200 to 28–30 (approximately 1-in-1 to 1-in-7, well within the boundary for maintaining accuracy [11]). Supratentorial and infratentorial volumes were manually delineated in the OsiriX software using the UCLResearchVolumes Plugin. The mid-sagittal slice was delineated first due to the robust and easily viewable anatomical landmarks. This then guided the use of anatomical landmarks in parasagittal slices. In the midline, the infratentorial volume was considered the space enclosed by a line along the tentorium cerebelli, a line from the free edge of tentorium cerebelli (at the tentorial notch) to the dorsum sellae, a line from the dorsum sellae down the clivus to the basion, McRae’s line from the basion to the opisthion and a line from the opisthion to the torcular shelf. The supratentorial volume was considered the space enclosed by a line across the tentorium cerebelli, a line around the inner table of the neurocranium, including the sella turcica (the pituitary volume was counted within the supratentorial volume), and a straight line to the tentorial notch*.*

Aware of potential learning bias, all scans were subject to a final re-review before statistical analysis.

### Blinding

All reviewers, who independently determined PCD status, were blinded to each other’s analysis. Blinding the reviewer to PCD status when calculating ICV was not technically possible as the reviewer who calculated ICV also reviewed PCD status. However, volume scans were randomised prior to analysis to mitigate this. Furthermore, determining PCD status from imaging is dependent on a mid-sagittal slice. A mid-sagittal slice was rarely obtained due to image series being generated from between the image’s two lateral boundaries.

### Statistical analysis

The null hypothesis stated there is no statistically significant difference between supratentorial and infratentorial PCD for the increase of ICV per mm distraction. IBM SPSS 25 was used to analyse the dataset from this cohort. A *P* value of < 0.05 was considered significant (i.e. *P* = 0.05 was not statistically significant). Student’s *t* test was used to compare supratentorial and infratentorial data. IBM SPSS 25 was also used to calculate intraclass correlation coefficient (ICC) for determining interchangeability the CT and MRI scans for calculating ICV.

## Results

Our cohort comprised 20 cases of supratentorial and 27 cases of infratentorial PCD. There were no statistically significant differences between the two sub-cohorts, other than the period that elapsed between the pre-operative CT images and surgery; in the supratentorial group, the average period was 86.7 days (SD 78.8), compared to 189.9 days (SD 169.8) in the infratentorial group (*P* = 0.045) (Table 1).

**Table 1:** Comparative characteristics between the supratentorial and infratentorial cohorts of this study

Demographic	Supratentorial PCDO	Infratentorial PCDO	*P* value
Participants	20	27	
Average age at operation, months (SD)	45.6 (60.1)	37.8 (42.5)	0.623
Male (%)	12 (60.00)	14 (51.85)	
Female (%)	8 (40.00)	13 (48.15)	
Average pre-op scans to op delay - CT, days (SD)	86.7 (78.8)	189.9 (169.8)	0.045
Average op to post-op scans delay - CT, days (SD)	340.3 (204.9)	417.6 (222.2)	0.362
Average pre-op scans to op delay - MRI, days (SD)	154.5 (106.7)	129.4 (94.6)	0.484
Average op to post-op scans delay - MRI, days (SD)	527.1 (378.2)	489.7 (292.4)	0.757
Average distraction distance, mm (SD)	24.2 (7.7)	24.4 (4.9)	0.881
Evidence of raised ICP (%)	17 (85.00)	21 (77.78)	
Pansynostosis (%)	11 (55.00)	11 (40.74)	
Multi-suture synostosis (%)	1 (5.00)	1 (3.70)	
Bi-coronal synostosis (%)	7 (35.00)	10 (37.04)	
One suture synostosis (%)	0 (0)	5 (18.52)	
Confirmed synostotic syndrome (%)	13 (65)	9 (33.33)	

The primary outcome for comparing supratentorial and infratentorial PCD was percentage volume increase per mm distraction. Combined CT and MRI data is presented for comparing infratentorial and supratentorial PCD for the increase of STV, ITV and total ICV.

### Supratentorial volume

**Fig. 2:**
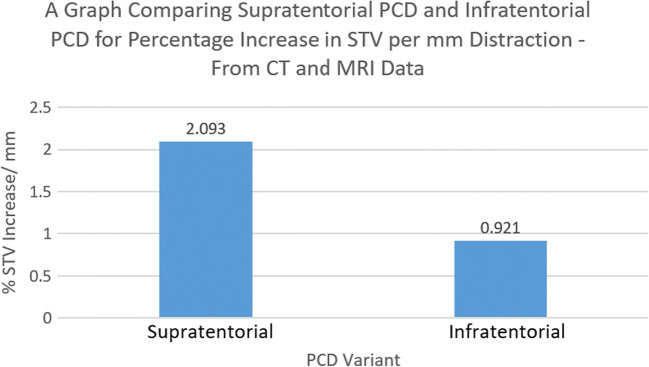
A bar chart comparing supratentorial and infratentorial PCD for the increase of supratentorial volume—calculated from both CT and MRI data

The combined CT and MRI data showed that supratentorial PCD was more effective than infratentorial PCD at increasing supratentorial volume; this was statistically significant at the 95% confidence level. The mean increase in STV, as a percentage of pre-operative volume, per millimeter distraction was 2.093% for supratentorial PCD compared to 0.921% for infratentorial PCD (*P* = 0.0458) (Fig. 2).

**Fig. 3:**
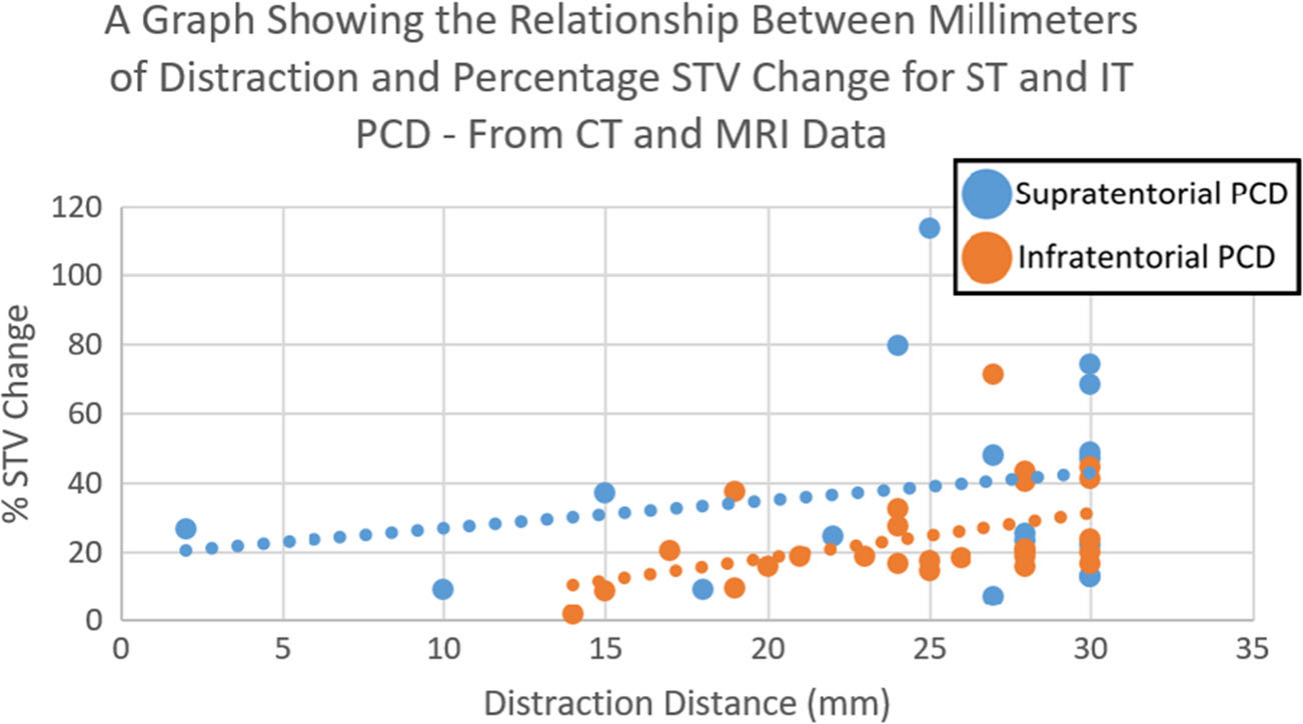
A scatter graph comparing supratentorial and infratentorial PCD for the increase of supratentorial volume for a given distance of distraction—calculated from CT and MRI data

Further analysis of this data indicated that the difference was not constant across all distraction distances, though supratentorial PCD was more effective than infratentorial PCD at all distraction distances with a trend towards convergence beyond the physical limit of 30 mm imposed by the distractors (Fig. 3).

### Infratentorial volume

**Fig. 4:**
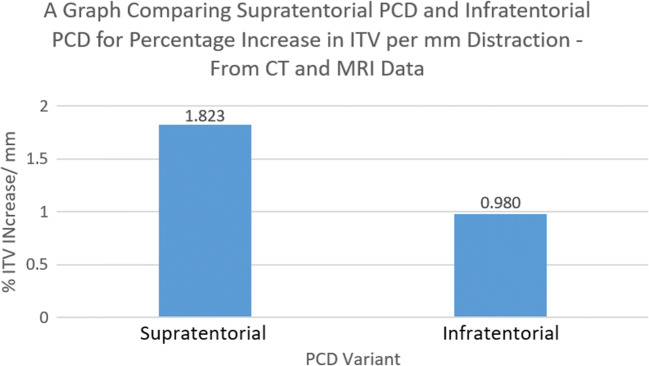
A bar chart comparing supratentorial and infratentorial PCD for the increase of infratentorial volume—calculated from combined data

The combined CT and MRI data showed that supratentorial PCD was more effective than infratentorial PCD in increasing ITV per millimeter distraction, though this was not statistically significant at the 95% confidence level. The combined data showed that the mean percentage volume increase per millimeter distraction for supratentorial PCD was 1.823%, compared to 0.980% for infratentorial PCD (*P* = 0.0697) (Fig. 4).

**Fig. 5:**
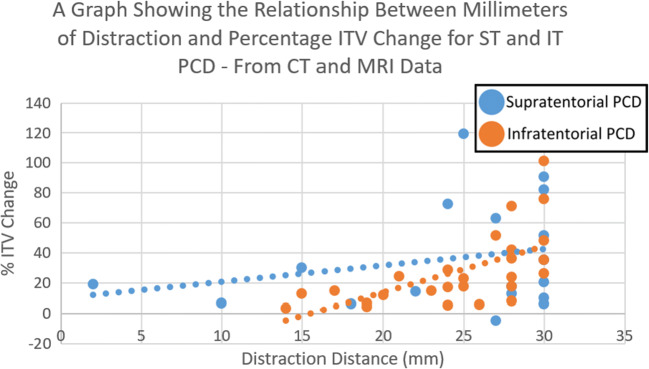
A scatter graph comparing supratentorial and infratentorial PCD for the increase of infratentorial volume for a given distance of distraction—calculated from combined data

Further analysis of this data exposed that this difference was not constant across all distraction distances, with infratentorial PCD becoming more effective than supratentorial PCD at between 29 and 30 mm distraction (Fig. 5).

### Total intracranial volume

**Fig. 6:**
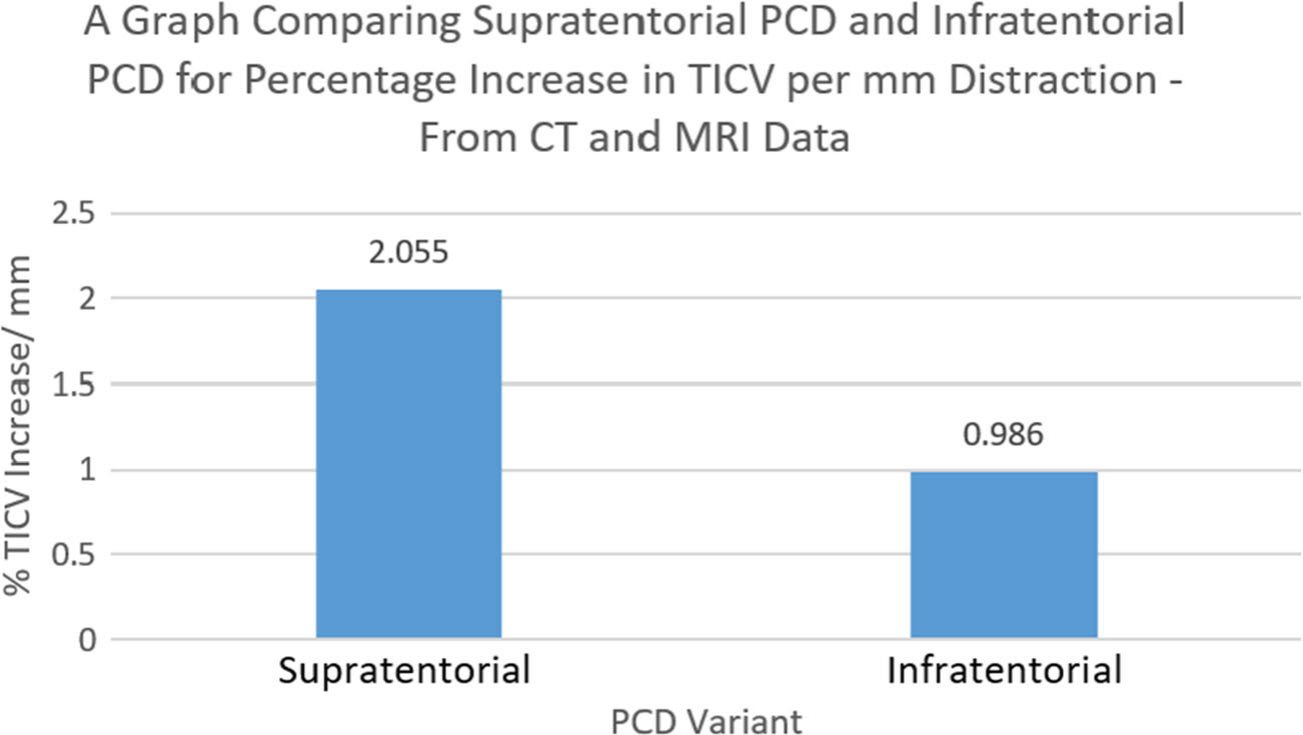
A bar chart comparing supratentorial and infratentorial PCD for the increase of total intracranial volume—calculated from CT and MRI data

Analysis of combined CT and MRI data showed that supratentorial PCD was more effective than infratentorial PCD at increasing total intracranial volume per millimeter distraction; this was statistically significant at the 95% confidence level. The mean TICV percentage increase per mm distraction for supratentorial PCD was 2.055% compared to infratentorial PCD which only elicited 0.986% (*P* = 0.0437) (Fig. 6).

**Fig. 7:**
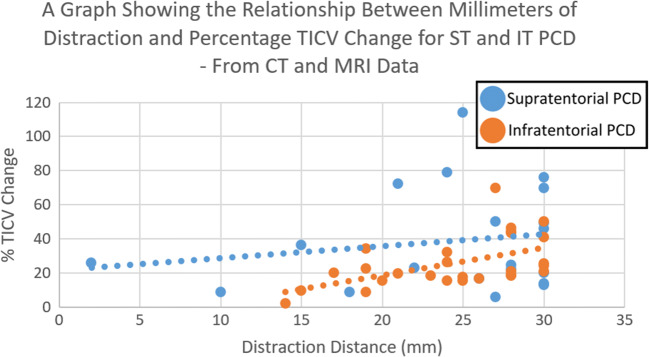
A scatter graph comparing supratentorial and infratentorial PCD for the increase of total intracranial volume for a given distance of distraction—calculated from combined data

Further analysis of this data showed that this difference varied across distraction distances, though the data shows a trend towards convergence beyond the physical limit of 30 mm imposed by the distractors (Fig. 7).

### Clinical interchangeability of CT and MRI for ICV measurement

Intraclass correlation coefficient (ICC) was used to assess the agreeability of CT and MRI imaging modalities for measuring ICV. ICC for CT and MRI estimating STV was 0.852, for estimating ITV ICC was 0.864 and for TICV ICC was 0.854.

## Discussion

This study compared supratentorial and infratentorial PCD for the increase of ICV compartments to determine which was more effective. The secondary aim of this study was to assess the agreeability of CT and MRI modalities for estimating ICV.

### Outcomes

These data suggest that the surgical consensus—infratentorial PCD is more effective than supratentorial PCD for increasing ICV in craniosynostosis—may be incorrect. Supratentorial PCD was shown to be more effective than infratentorial PCD for increases in ICV, with the relative increases in STV and TICV being statistically significant. The lines of best fit did tend towards convergence, though in the case of STV and TICV increases this convergence was not seen before the distractors’ physical limit of 30 mm. Some cases did not complete the full distraction protocol to 30 mm due to either trauma to the distractors, CSF leak, wound infection, tissue necrosis or re-fusion of the osteotomy—this was not associated with procedure variant.

### Mechanism of effect

**Fig. 8:**
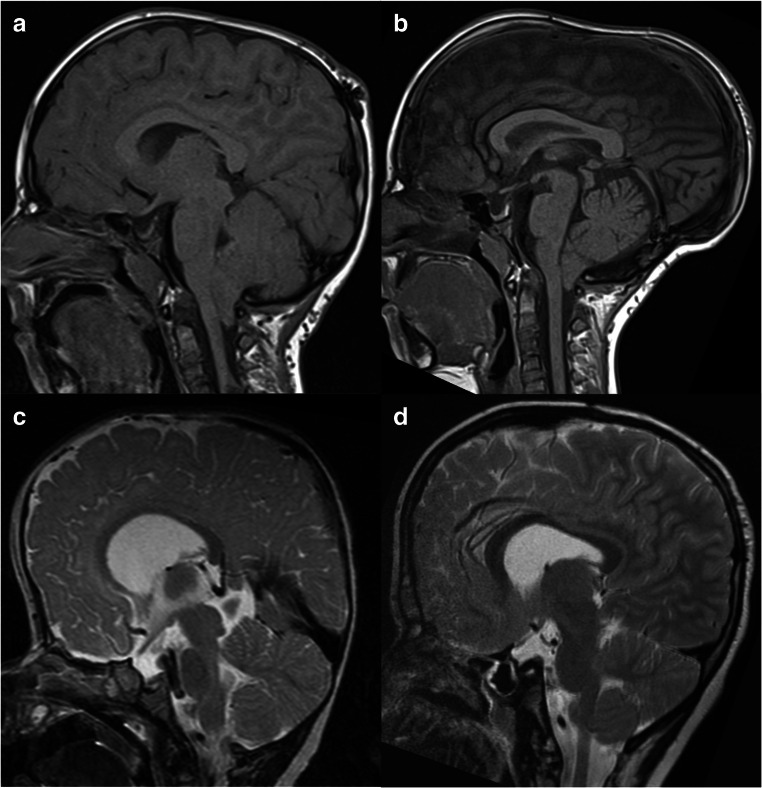
Pre-op and post-op mid-sagittal MRI images from a case of supratentorial (**a**, **b**) and infratentorial (**c**, **d**) PCD, from this study. Panels **a** and **c** show pre-op imaging for a supratentorial and infratentorial case respectively. Panels **b** and **d** show post-op imaging for the same supratentorial and infratentorial cases respectively. Pre-op images are from within 1 year of the operation date; post-op images are from at least 500 days post-op, when bone healing was certainly complete. Note in panel **d** the flattening of the tentorium, and compression/smoothing of the cerebellum, relative to the pre-op image in panel **c**—a change not seen between panels **a** and **b**, where the tentorium rises post-operatively (note the angles between the inner table and the superior aspect of the tentorium)

Suggesting infratentorial PCD was inherently more effective at increasing ICV compared to supratentorial PCD was based on a greater surface area of mobile calvarium being distracted in infratentorial PCD. We have shown this assumption may be incorrect. We suggest that the reason for this is that infratentorial PCD mobilizes the posterior-most attachment of the tentorium cerebelli (the torcular shelf). Subsequently, as the distraction progresses, the tentorium cerebelli is put under tension (owing to its intact anterior attachments [8]) and flattens, thereby compressing the infratentorial space (Fig. 8). Therefore, increases in the STV are mitigated against losses of the ITV in infratentorial PCD. Supratentorial PCD avoids mobilizing the tentorium, and subsequently decompresses the STV whilst allowing the tentorium to rise into the STV, decompressing the ITV also. The literature identified throughout this study rarely recognized supratentorial PCD as a surgical option. We believe these results should be taken into account when planning future cases of PCD. Further research, ideally with larger cohorts, is required to corroborate these findings.

### Application to practice

Intraoperatively, the tentorium was identified by measuring the distance between the foramen magnum and torcular shelf using CT imaging, and then manually applying this to the patient’s skull. These results suggest that supratentorial PCD may be considered for cases of multi-suture or syndromic craniosynostosis, over infratentorial PCD. This involves decreasing the angle between osteotomies at the asterion to ensure the inferior osteotomy always remains above the anatomical attachments of the tentorium cerebelli in the temporal and occipital bones.

Though not conducted in this study, and only reported in infratentorial PCD, evidence suggests that barrel stave osteotomies, with absorbable plates, may confer benefit to surgical outcomes by minimising the ‘step’ in the posterior occiput, sometimes caused by supratentorial PCD [12]. Though this may put the tentorium under tension.

### Clinical interchangeability of CT and MRI for ICV estimation

ICC was used to assess the agreeability of CT and MRI imaging modalities for estimating ICV to review if unnecessary radiation exposure in craniosynostotic children could be avoided. The ICC values for CT and MRI in assessing STV, ITV and TICV were 0.852, 0.864 and 0.854 respectively. Literature published on the interpretation of ICC shows that these data represent good, bordering on excellent, agreement [13]. Subsequently, for assessing ICV, CT and MRI scans are clinically acceptably similar. We assessed T1 and T2 MRI together. MRI scans tended to overestimate ICV compared to CT, potentially due to the relative lack of brain-bone contrast, especially in T1-weighted scans. This could be investigated by assessing T1-weighted and T2-weighted MRIs separately.

### Limitations of this study

#### Missing data

This study recruited 47 cases eligible for the final analysis—one of the largest cohorts in the country. A factor in excluding 25 cases from the study was a lack of post-operative scans in the most recent 12 cases (from March 2017 onwards). Despite no changes in practice from March 2017, it is worth commenting that missing data disproportionately affected more recent cases which did not have a clinical need to be scanned. However, it is likely that these patients would be scanned at some point in the future. Subsequently, it must be mentioned that there is a potential that the more recent excluded patients were significantly different from those patients kept in the study. This said, some post-operative follow-up times exceeded the time that elapsed between March 2017 and October 2018 (the start of data collection), so this may not be the case.

#### Follow-up times and variable patient ages

There was some variation between cases regarding the elapsed period between pre-operative images, surgery and post-operative images. This likely would not be a problem in an adult population due to halted calvarial growth; however, for this paediatric population, the highly variable rate of skull growth in the first 3 years of life makes it difficult to account for skull growth between pre-operative images and surgery [14, 15]. However, the infratentorial group saw a greater delay between pre-op scans and surgery which would have allowed more time for growth and potentially contributed to the expansion attributed to the infratentorial PCD procedure—this study still elucidated supratentorial PCD may be a more effective surgery so this statistically significant difference in delays may only serve to underestimate the magnitude of the trends shown here. This is not a significant issue for the post-operative imaging delay since calvarial surgery tends to halt skull growth [16, 17]. A compounding factor of this is the variable age of the patients; the rate of calvarial growth is not constant over the first 3 years of life, nor does it vary uniformly [18]. Using standardized skull growth charts to infer the rate of skull growth for patients of different ages would not be robust. Regardless, we do not anticipate that this factor significantly affected the results due to lack of a statistically significant difference between the two groups with regard to age. Due to late referrals and one case of prior calvarial surgery, some cases are older than would be expected for this operation. However, owing to the mechanical nature of the intervention, the authors do not believe that this significantly affects the applicability of the results.

### Drawbacks of this methodology

#### Determining PCD status prior to ICV delineation

PCD status was determined before ICV calculation. This may have introduced an information bias but was mitigated by two factors: (1) the randomised order in which the 47 cases were approached; (2) the operation to post-op imaging period was often long enough to ensure bone regrowth across the osteotomies so PCD status could not be inferred from post-op scans. We do not think that this affected results.

#### One case of reduced ITV

One case of supratentorial PCD saw a reduction in ITV despite 27 mm distraction. This case was recalculated with a consultant interventional radiologist at BCH to assess for methodological error. The recalculation confirmed there was no methodological error and that the scans reliably indicate a genuine reduction in ITV in this case. Though not fully elucidated, this may be due to decompressive restructuring of the supratentorial contents in combination with significant unique anatomical aberration of the posterior cranial fossa.

## Conclusion

We have shown that supratentorial PCD may be more effective than infratentorial PCD for increasing ICV in children with syndromic, or extensive, craniosynostosis. We elucidated statistically significant differences between supratentorial and infratentorial PCD for increasing STV and TICV, in favour of supratentorial PCD. We believe this evidence should be considered in surgical planning of future cases of syndromic craniosynostosis. Furthermore, we found good agreeability between CT and MRI imaging modalities for estimating ICV in children with craniosynostosis, which may be justification to spare children radiation from CT scanning for this purpose.

## Data Availability

NA.
